# Migrants’ motives and expectations for contacting out-of-hours primary care: a survey study

**DOI:** 10.1186/s12875-017-0664-7

**Published:** 2017-11-21

**Authors:** Ellen Keizer, Peter Bakker, Paul Giesen, Michel Wensing, Femke Atsma, Marleen Smits, Maria van den Muijsenbergh

**Affiliations:** 1 Scientific Center for Quality of Healthcare (IQ healthcare), Radboud Institute for Health Sciences, Radboud University Medical Center, P.O. Box 9101, 6500 HB Nijmegen, The Netherlands; 2 Department of Primary and Community Care, Radboud Institute for Health Sciences, Radboud University Medical Center, P.O. Box 9101, 6500 HB Nijmegen, The Netherlands; 3 Pharos, Centre of Expertise of Health Disparities, P.O. Box 13318, 3507 LH Utrecht, The Netherlands

**Keywords:** After hours care, Primary health care, Motivation, Expectations, Migrants

## Abstract

**Background:**

Migrants are more likely to use out-of-hours primary care, especially for nonurgent problems. Their motives and expectations for help-seeking are as yet unknown. The objective of this study is to examine the motives and expectations of migrants for contacting out-of-hours primary care.

**Methods:**

We used data from a survey study of 11,483 patients who contacted a General Practitioner (GP) cooperative in the Netherlands between 2009 and 2014 (response rate 45.6%). Logistic regression analysis was used to test differences in motives and expectations between non-western and western migrants and native Dutch patients.

**Results:**

The main motives for contacting a GP cooperative for non-western and western migrants were an urgent need for contact with a GP (54.9%–52.4%), worry (49.3%–43.0%), and a need for medical information (21.3%–26.2%). These were also the most important motives for native Dutch patients. Compared to native Dutch patients, non-western migrants more often perceived an urgent need for a GP (OR 1.65; 99% CI 1.27–2.16), less often needed information (OR 0.59; 99% CI 0.43–0.81), and more often experienced problems contacting their own GP during office hours (OR 1.71; 99% CI 1.21–2.43). Western migrants also reported experiencing problems more often in contacting their own GP (OR 1.38; 99% CI 1.04–1.84).

As well as for natives, most non-western and western migrants expected to see a doctor (46.2%–46.6%) or get advice (39.6%–41.5%). Non-western migrants expected more often to get physical examination (OR 1.53; 99% CI 1.14–2.04), and prescription (OR 1.37; 99% CI 1.00–1.88). We found no differences in expectations between western migrants and native Dutch patients.

**Conclusion:**

The main motives and expectations of migrants are similar to native Dutch patients, yet non-western migrants more often wanted action from the GP, e.g. examination or prescription, and less often passive forms of assistance such as giving information. At the same time they experience problems accessing their own GP. We recommend stimulation of self-care, education about the purpose of a GP cooperative, and examination and improvement of accessibility of daytime primary care.

## Background

There is a worldwide increase in migration flows between countries [1]. The Netherlands has also experienced an increase in the number of migrants. In 2015, 3.7 million inhabitants were migrants or children of migrants, which constituted 22% of the Dutch population [2]. Of these migrants, 56% have a non-western background and 44% a western background. By 2060, the proportion of these migrants is expected to have increased up to 31% [3].

Migrants’ utilisation of healthcare services has often been investigated, showing a variety of results. Most studies found that migrants have a relatively high use of both primary care and some types of specialist care, while preventive care and other forms of healthcare are used less frequently [4–7]. Studies also showed differences in healthcare use between various migrant groups [8, 9], most notably between migrants with a non-western and a western origin. Non-western migrants also appear to have a higher use of primary healthcare services compared to western migrants. The demand of out-of-hours primary care turned out to be higher in deprived areas, where a relatively higher number of non-western migrants reside [10]. Moreover, general practices with a larger number of migrants in their patient population were found to have higher out-of-hours primary care use [11].

Previous studies at the Emergency Department (ED) and out-of-hours primary care showed that migrants more often use care for problems that are unnecessary from a medical perspective [12–14]. A possible explanation is that non-western migrants differ in help-seeking behaviour and have other expectations of healthcare than native citizens, because they are used to a different healthcare system in their country of origin, have a poorer health status [15–17] and have lower health literacy skills [18, 19]. Another reason could be linked to differences in the perception of urgency. Problems that may well be considered as nonurgent in the Netherlands, are often considered as urgent in other countries. This is likely to be caused by higher morbidity and mortality rates from infectious diseases in non-western countries compared to western countries [20].

In the Netherlands, each patient has to be registered in a general practice of his own choice, with general practitioners (GP) acting as gatekeepers for secondary care. Referrals are needed for visits to medical specialists in hospitals and are initiated and coordinated by the GP [21]. Migrants with residence permit have the same entitlements to GP care as all other Dutch people. Out-of-hours primary care is provided by 120 large-scale GP cooperatives [22]. The cooperatives serve 99% of the Dutch population of 17 million and are available every evening, night, weekend and during the holidays. Each GP cooperative has 50 to 250 GPs who provide care to 100,000 to 500,000 citizens. Every GP has to do a minimum number of shifts at the GP cooperative to maintain his/her registration as a GP. Patients are classified in urgency categories from high to low urgency. Key features of GP cooperatives in the Netherlands are listed in Table 1.

**Table 1:** Features of general practitioner (GP) cooperatives in the Netherlands and charging system [22, 23, 44]

Theme	Feature
General	Out-of-hours primary care has been provided by large-scale general practitioner (GP) cooperatives since the year 2000
	Every GP has to do a minimum number of shifts at the GP cooperative to maintain his/her registration as a GP.
	Participation of 50–250 GPs per cooperative with a mean of 4 h on call per week with a compensation of about €65/h
	About 120 GP cooperatives in the Netherlands
	Population of 100,000 to 500,000 patients with an average care consumption of 250 contacts/1000 inhabitants per year
	Out-of-hours defined as daily from 5 p.m. to 8 a.m., all public holidays and the entire weekend
	Per shift GPs have different roles: supervising telephone triage, doing centre consultations or home visits
	The triage is supervised by telephone consultation doctors who can be consulted in case of doubt, while also checking and authorising all calls
Location	GP cooperative usually situated in or near a hospital
	Distance of patients to GP cooperative is 30 km at most
Accessibility	Access via a single regional telephone number, meaning the first contact is mostly with a triage nurse (only 5–10% walk in without a call in advance)
	Telephone triage by nurses supervised by GPs: contacts are divided into telephone advice (38%), centre consult (52%), or GP home visit (9%)
Facilities	Home visits are supported by trained drivers in identifiable fully equipped GP cars (e.g. oxygen, intra venous drip equipment, automated external defibrillator, medication for acute treatment)
	Information and communication technology (ICT) support including electronic patient files, online connection to the GP’s car, and sometimes connection with the electronic medical record in the GP’s daily practice
Charging system	Healthcare is largely covered by health insurance
	All residents over 18 years pay a monthly premium to their health insurance provider. There is no premium for children
	Employers pay a part of their employee’s income to the tax administration for healthcare costs
	Patients do not have to pay an additional amount for GP care, both during and outside office hours
	Residents over 18 years must an annual deductible (385 euro in 2016) in case of use of healthcare (including emergency departments). This deductible is neither applicable to GP care, nor to children

The GP cooperative is intended for urgent help requests that cannot wait until the regular consultation hours of the patient’s own GP. However, in practice, a large part of the help requests proved to be nonurgent from a medical perspective (45%) [23]. As (non-western) migrants are more likely to use care for nonurgent problems, gaining insight into the motives and expectations of migrants for contacting GP cooperatives can be of value.We expect that their motives are often worry and a perceived need to see a doctor for a physical examination, because of the differences in contextual circumstances in their country of origin, such as morbidity and mortality from infections. Previous research has also shown that migrants more often perceive problems with their own GP’s accessibility [24]. Therefore, low accessibility to daytime general practice could also be a motive for contacting out-of-hours primary care.

Since the migrant population is increasing, it is clearly relevant to understand the motives and expectations of this particular population. The objective of our study is to examine the motives and expectations of migrants for contacting out-of-hours primary care.

## Methods

### Design, setting and population

We used an existing dataset of survey studies on patient experiences, which we performed in stratified samples of patients who contacted a GP cooperative between 2009 and 2014. Data from 11,483 patients were available (response rate 45.6%). Stratification was based on the type of contact: equal numbers of questionnaires were distributed to patients who only had a telephone contact, patients who had a GP consultation at the GP cooperative, and patients who had a GP home visit. Data from a convenience sample of 42 GP cooperatives (from a total of approximately 120) spread across the Netherlands were used.

### Questionnaire

For our study, we used the Consumer Quality Index (CQI) GP cooperatives [25]. This Dutch questionnaire was developed by the department of IQ healthcare of the Radboud University Medical Center Nijmegen and validated in the general population of the GP cooperative [26]. The questionnaire included questions about patient characteristics, expectations of healthcare, motives for seeking healthcare, and patient experiences in healthcare. In this study we used only a part of the questionnaire, namely the motives and expectations for contacting the GP cooperative as well as the origin of the patient and his/her parents. Patients had to indicate for each motive and expectation whether it actually applied to them. These motives and expectations were the outcome measures in our study. Age, gender, education and health status of patients were used for case-mix adjustment in data analysis. Health status was measured with a 5-point Likert scale by asking patients to describe their own health (very good, good, fair, bad, very bad).

### Procedure

At each GP cooperative, a representative sample of 600 patients received the questionnaire by post in a four-week period from 2009 to 2014. Patients received the questionnaire between four and ten days after their contact with the GP cooperative, while a reminder was sent after one week and after three weeks.

We asked the parents of patients aged under 12 to fill in the questionnaire. The following exclusion criteria were used: dying or deceased patients; patients who contacted the GP cooperative for administrative reasons or for confidential problems; patients residing abroad; exceptional telephone stalkers (calling several times without a help request) and patients who declared not to be willing to participate in research.

The three questions in the questionnaire about the origin of the patient, his/her father and his/her mother were used to determine whether a patient was a western migrant, a non-western migrant or a native Dutch patient. Migrants were defined in accordance with Statistics Netherlands, meaning that at least one parent was born abroad [27]. The patients were divided into three groups: non-western migrants (originating from Africa, Latin America, Asia -except Indonesia and Japan- or Turkey), western migrants (originating from European countries -except Turkey-, North America and Oceania, Indonesia or Japan) and native Dutch patients (both parents born in the Netherlands). If the parents were born in different countries outside the Netherlands, we used the mother’s country of birth to determine the patient’s origin. When the country of birth of the parents was unknown, we used the country of birth of the patient to define the origin. If the parents completed the questionnaire for their child we used their data to determine the origin.

### Statistical analysis

Missing data occurred on the outcome variables motives (*N* = 416; 3.6%) and expectations (*N* = 155; 1.3%), as well as origin (*N* = 629; 5.5%), gender (*N* = 1060; 9.2%), age (*N* = 1050; 9.1%), education (*N* = 1321; 9.1%) and self-reported health status (*N* = 1544; 13.4%). Results from a MNAR (missing not at random) test showed that the missing data appeared to be at random. We used multiple imputation (MI) to impute missing values (five imputation sets).

Descriptive statistics were used to describe patient characteristics, motives and expectations for contacting the GP cooperative. For each motive and expectation, differences between migrants and natives were tested with logistic regression analysis, while pooled odds ratios were calculated (pooling of all odds ratios of the single imputation sets into one overall odds ratio). To account for clustering of patients within GP cooperatives, the variable GP cooperative was added as a covariate in the analyses. In the analyses, we corrected for age, gender, education and self-reported health status, as these patient characteristics might influence the response tendencies of the respondents [28, 29]. Differences between migrants and natives were tested for motives and expectations with sufficient response heterogeneity (at least 5% /95%). This means that enough discrimination between the yes and no group was needed. Models were constructed for the following motives: urgent need for a GP, worry, need for medical information, own GP could not be contacted during office hours, and for all expectations. In order to account for multiple testing, we used *p* < 0.01 (99% CI) to determine the significance of the differences between the groups of origin.

Goodness of fit between the observed and predicted outcomes of the logistic models were assessed based on the Hosmer-Lemeshow test and its discrimination ability was assessed based on the area under the receiver operating curve (AUC). Since it is not possible in SPSS to pool the Hosmer-Lemeshow test and the AUC results, we presented the results of one single imputation set. Data were analysed using the Statistical Package for the Social Sciences (SPSS) 22.

## Results

### Multiple imputation

After multiple imputation of our data, we compared the descriptive statistics of the patient characteristics, motives and expectations of the original data with the pooled data. As the results were almost similar, we decided to present the results of the pooled data.

### Patient characteristics

Table 2 shows a description of the study population for the different groups of origin. Of the respondents, 4.1% (*N* = 475) were non-western migrants, 6.1% (*N* = 700) western migrants and 89.8% (*N* = 10,308) native Dutch patients. We noticed a few minor differences between the origins of the groups regarding distribution in gender, education and self-reported health status. Compared to native Dutch patients, the non-western migrants in our sample seemed younger.

**Table 2:** Description of study population (%)

Characteristic	Non-western (*N* = 475)	Western (*N* = 700)	Native dutch (*N* = 10,308)
Gender			
Male	39.9	40.1	42.9
Female	60.1	59.9	57.1
Age groups			
0–4	11.7	5.8	8.2
5–17	36.6	17.4	18.3
18–44	36.4	42.1	36.5
45–64	9.1	24.0	25.3
≥ 65	6.2	10.7	11.7
Education			
Low (≤ 10 years education)	38.2	35.8	42.7
Medium (11–14 years education)	38.2	33.4	32.4
High (≥ 15 years education)	23.6	30.8	24.9
Self-reported health status			
Excellent / very good	26.2	35.0	37.1
Good	46.7	37.2	38.8
Moderate / poor	27.1	27.8	29.4

### Motives for seeking healthcare

The most frequently mentioned motives for both non-western and western migrants to contact a GP cooperative were urgent need for a GP (54.9% - 52.4%), worry (49.3% - 43.0%) and need for medical information (21.3% - 26.2%) (Table 3). These motives were also most often mentioned by native Dutch patients. We found some minor differences between migrants and native Dutch patients (Table 4). Non-western migrants more often perceived an urgent need for contact with a GP as opposed to native Dutch patients (OR 1.65, 99% CI 1.27–2.16, corrected for gender, age, education and self-reported health status). They less often mentioned a need for medical information as a motive for contacting a GP cooperative (OR 0.59, 99% CI 0.43–0.81). They also reported more often that they could not contact their own GP during office hours (OR 1.71, 99% CI 1.21–2.43). Western migrants also mention more often that they could not contact their own GP during office hours (OR 1.38, 99% CI 1.04–1.84). We found no other differences in motives between western migrants and native Dutch patients. All four logistic models achieved sufficient calibration (Hosmer-Lemeshow test’s *P* range of 0.129–0.171 in a single imputation set) and discrimination (AUC range of 0.606–0.651 in a single imputation set).

**Table 3:** Motives and expectations of patients for contacting a GP cooperative (%)

	Non-western (*N* = 475)	Western (*N* = 700)	Native dutch (*N* = 10,308)
Motive^1^			
I urgently needed a GP	54.9	52.4	48.2
I was worried	49.3	43.0	45.3
I needed medical information	21.3	26.2	27.2
My own GP could not be contacted during office hours	18.7	15.7	12.2
I had been referred to the GP cooperative by another caregiver	3.6	3.5	4.6
I did not have time to go to the GP during the day	3.3	1.9	1.3
The ED was not prepared to help me	3.0	2.1	0.8
I needed a second opinion	2.6	1.1	0.8
Expectation^1^			
Seeing a doctor	46.2	46.6	44.5
Advice	39.6	41.5	39.7
Physical examination	27.4	18.6	19.3
Prescription or medication	24.5	20.3	17.1
Reassurance	24.0	19.8	16.6
Referral to a hospital	12.2	13.2	12.0
Treatment (e.g. a stitch)	7.4	7.4	7.5

^1^Multiple answers were possible

**Table 4:** Logistic regression of motives and expectations of patients for contacting a GP cooperative (*N* = 11,483)

	OR Non-western^a, b^ (99% CI)	OR Western^a, b^ (99% CI)
Motive		
I urgently needed a GP	1.65 (1.27–2.16)*	1.13 (0.91–1.41)
I was worried	0.96 (0.73–1.28)	0.92 (0.74–1.14)
I needed medical information	0.59 (0.43–0.81)*	0.96 (0.74–1.25)
My own GP could not be contacted during office hours	1.71 (1.21–2.43)*	1.38 (1.04–1.84)*
Expectation		
Seeing a doctor	1.23 (0.96–1.59)	1.09 (0.87–1.35)
Advice	0.79 (0.59–1.07)	1.09 (0.87–1.35)
Physical examination	1.53 (1.14–2.04)*	0.94 (0.71–1.23)
Prescription or medication	1.37 (1.00–1.88)*	1.22 (0.94–1.59)
Reassurance	1.34 (0.96–1.85)	1.28 (0.99–1.67)
Referral to a hospital	1.28 (0.87–1.90)	1.10 (0.81–1.49)
Treatment (e.g. a stitch)	1.00 (0.60–1.67)	0.98 (0.64–1.50)

*OR* Odds Ratio, *CI* Confidence Interval

^a^Reference category: Native Dutch patients

^b^Corrected for gender, age, education and self-reported health status

^*^ *p* < 0.01, in bold

### Expectations of healthcare

The expectations most often mentioned for both non-western and western migrants were seeing a doctor (46.2% - 46.6%) and getting advice (39.6% - 41.5%) (Table 3). Native Dutch patients also mentioned these expectations most often. A smaller group of non-western migrants expected to get a physical examination (27.4%), or expected prescription or medication (24.5%), or to be reassured (24.0%). A small group of non-western and western patients expected to be referred to a hospital (12.2% - 13.2%) or to receive treatment (e.g. a stitch) (both 7.4%). There were two significant differences between non-western migrants and native Dutch patients and no differences between western migrants and native Dutch patients (Table 4). Non-western migrants more often expected get a physical examination (OR 1.53, 99% CI 1.14–2.04) and to get a prescription or medication (OR 1.37, 99% CI 1.00–1.88) compared to native Dutch patients. All seven logistic models achieved both calibration (Hosmer-Lemeshow test’s mean *P* range of 0.173–0.966 in a single imputation set) and discrimination (AUC range of 0.576–0.664 in a single imputation set).

### Subgroup analyses

Due to a relatively small number of migrants, we showed the results for two main groups of migrants: non-western and western. These two groups being heterogeneous, we performed subgroup analyses for the largest countries of origin. We found no major differences in motives and expectations between the countries.

## Discussion

### Principal findings and interpretation

The most important motives for contacting a GP cooperative were similar for both migrants and native Dutch patients, namely worry, an urgent need for a GP and a need for medical information. We also found some differences, especially between non-western migrants and native Dutch patients. Compared to native Dutch patients, non-western migrants more often perceived an urgent need for contact with a GP. On the other hand, they less often mentioned a need for medical information. This could be explained by the fact that non-western migrants may have lower health literacy skills, resulting in poorer knowledge of healthcare services [18] and not knowing when to contact a GP cooperative [19, 30].

Migrants also reported more often that they could not contact their own GP during office hours. The accessibility of daytime general practice could be worse for migrants, because they are residing more often in urban areas where telephone accessibility in daytime general practices is generally poorer than in rural areas [31]. Moreover, longer telephone waiting times in daytime primary care are known to be associated with a higher use of out-of-hours primary care [11]. In addition, due to language barriers, migrants may have poorer negotiation skills, leading to barriers of accessing daytime general practice [32, 33].

Regardless of origin, most patients expected to see a doctor or to get advice. Non-western migrants more often expected to get a physical examination or to get a prescription or medication as opposed to native Dutch patients. Whenever migrants went to a physician in their country of origin, they were used to getting a physical examination or prescription and were reassured by these actions [34]. This could be possibly account for these differences.

### Comparison with existing literature

To our knowledge, the motives or expectations of migrants for contacting a GP cooperative have not been studied previously. The most often mentioned motives for contacting a GP cooperative mentioned in our study are in accordance with a British study on motives of the general public for contacting a primary care out-of-hours service [35]. Moreover a Dutch study about non-urgent contacts found worry, the perceived need for urgent contact with a GP and the need for medical information as most important motives for contacting a GP cooperative [14]. The importance of a physical examination in medical encounters, especially with migrants, is known from national as well as international qualitative studies [34, 36, 37].

Our finding that migrants experience difficulties in the accessibility of their own GP is consistent with a British study, which found that patients in deprived areas perceived more difficulties in accessing their GP during consultation hours [32]. A Danish study found that migrants more often reported going to the ED because they could not contact a GP, or were not able to explain their problem on the telephone [33]. A Norwegian study reported that migrants were less likely to consider contacting a GP before attending the ED, because they thought it would take too long to make an appointment to consult a GP and they expected the ED to be better able to deal with their problem than a GP [38].

### Strengths and limitations

A strength of our study is that we used a large dataset for this research, with a large group of patients from different GP cooperatives spread across the Netherlands. About one third of all Dutch GP cooperatives participated in this research. Our analyses were controlled for patient characteristics and cluster effects, while missing values had been imputated. The response rate was 45.9%, which is similar to response rates in other patient questionnaires in the setting of out-of-hours primary care services (39.7% to 45.7%) [39].

A limitation of our study is that we were not able to perform a non-response analysis; therefore it is difficult to determine whether our results are representative of all patients contacting a GP cooperative. The proportion of migrants was low compared to the proportion of migrants in the Dutch population, especially non-western migrants (4.1% versus 12.1%). Possibly, less integrated migrants were less likely to answer the questionnaire, due to language barriers [40]. Compared to the Dutch population, the distribution of the educational level of non-western migrants in our sample was the same. Even so, this is not representative of the whole non-western migrant community in the Netherlands (lower educational level) [41]. Moreover, it is unlikely that this is representative of the population who contacted a GP cooperative. This bias is due to the research method used: a written questionnaire in Dutch. Therefore, the migrants in our sample are not representative of all migrants in the Netherlands. Based on literature we can assume that lower educated non-western migrants face even more barriers in consulting their GP during daytime, due to limited health literacy [42]. In the analyses we corrected for self-reported health status, which is a subjective health measure reflecting a person’s general perception of health. However, self-reported health status is widely used in several studies, also among migrants, being a good predictor of objective health status [43].

The number of migrants being small, we showed the results for two main groups of migrants: non-western and western. We are aware that these are two heterogeneous groups. Subgroup analyses for the largest countries of origin in the Netherlands showed that no major differences existed between those countries. In our study we did not have information about the urgencies of the contacts, neither about the medical history of the patients. Therefore it was not possible to relate the motives and expectations to the medical urgency (according medical professionals) of the contacts, which may (partly) explain the differences in motives and expectation between non-western migrants and native Dutch patients.

### Implications for practice and future studies

Our results provide us with leads for practice and further research. It is important that migrants are informed about the healthcare system in the country in which they currently reside. Migrants are known not to be always very well-informed about the system [30]. Information on the purpose of the GP cooperative can be provided in general practices, during integration courses or in social meetings of migrants.

In addition, tailored communication by the patients’ own GPs is essential, breaking down possible language barriers and taking into account low literacy. GPs could examine the expectations of their patients during consultations, give self-care advice if possible and inquire whether their patients accept and understand the advice given. This can be combined with an explanation about the Dutch healthcare system. The accessibility of daytime general practice could be a subject of further study. We recommend to examine whether the daytime GP practice is less accessible for migrants, and if so, how this can be improved. We also recommend to study the health literacy of migrants to manage their (minor) problems at home, possibly with support of primary health care organisations. Their experiences can help other migrants to gain access to daytime general practice or to use self-care. For the future our advice would be to offer this questionnaire also orally and in other languages, to reach a larger and more representative group of migrants. Finally, an in-depth qualitative study could provide further insight into the motives, expectations, and circumstances that increase the likelihood of migrants accessing out-of-hours primary health care. Combining this qualitative information with our quantitative results could lead to more concrete recommendations for practice.

## Conclusion

Worry, the perceived need for a GP and for medical information are the most important reasons for contacting a GP cooperative, regardless of the patient’s ethnic background. Patients expect to see a doctor and to get advice. Compared to native Dutch patients, non-western migrants more often want their GP to undertake some kind of action, e.g. an examination or prescription, and not just provide passive forms of assistance such as medical information. At the same time, they experience problems accessing their own GP during office hours. We recommend the stimulation of self-care, educating migrants about the purpose of a GP cooperative and examining and improving access to daytime primary care, especially for migrants.

## Abbreviations

CI: Confidence interval
ED: Emergency department
GP: General practitioner
